# Supplementary material for: The Heat Stress Transcription Factor LlHsfA4 Enhanced Basic Thermotolerance through Regulating ROS Metabolism in Lilies (Lilium Longiflorum)
Source: Int J Mol Sci. 2022 Jan 5;23(1):572. doi: 10.3390/ijms23010572 (PMC8745440; doi:10.3390/ijms23010572)
Supplement: Supplementary file 1 [file ijms-23-00572-s001.zip › ijms-1481980-supplementary.pdf]

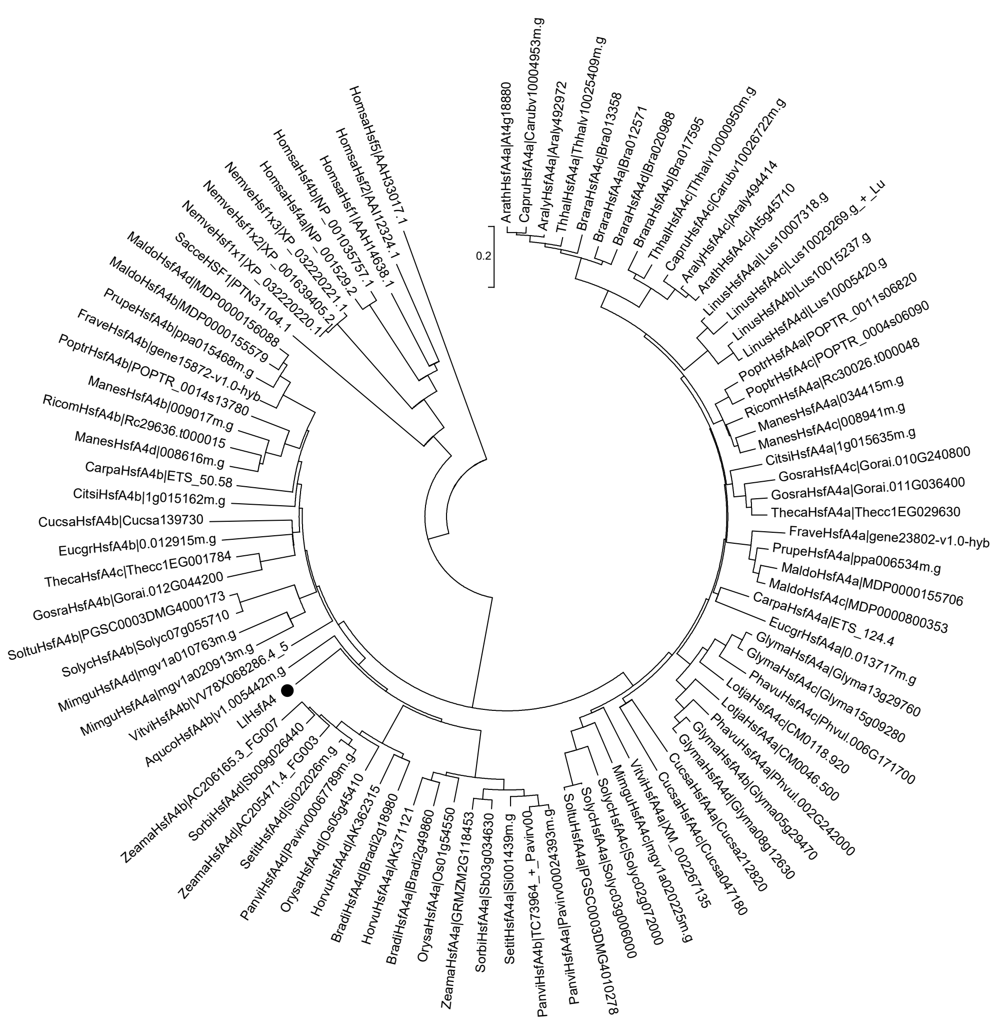

Supplementary Figure S1. Phylogenetic tree of LlHsfA4 and all Hsfs in different monocotyledonous plants and three non-plant organisms. This tree was constructed by ClustalW 2.0 and MEGA 5.0 software. The dark dot means LlHsfA4 protein.

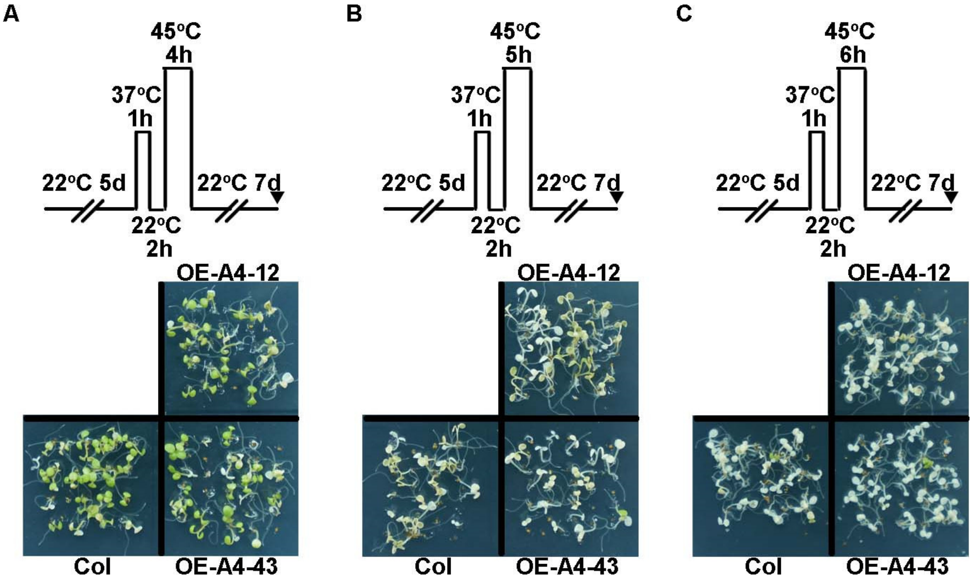

Supplementary Figure S2.Transgenic Arabidopsis plants of LlHsfA4 did not enhance aquired thermotolerance. 5-day old seedlings of two transgenic lines and wild type were treated with 37 ℃ for 1h ,then recovery for 2 h at 22℃,and then subjected to 45℃ with different times as 4 h (A),5 h (B) and 6 h (C) to examine acquired thermotolerance.

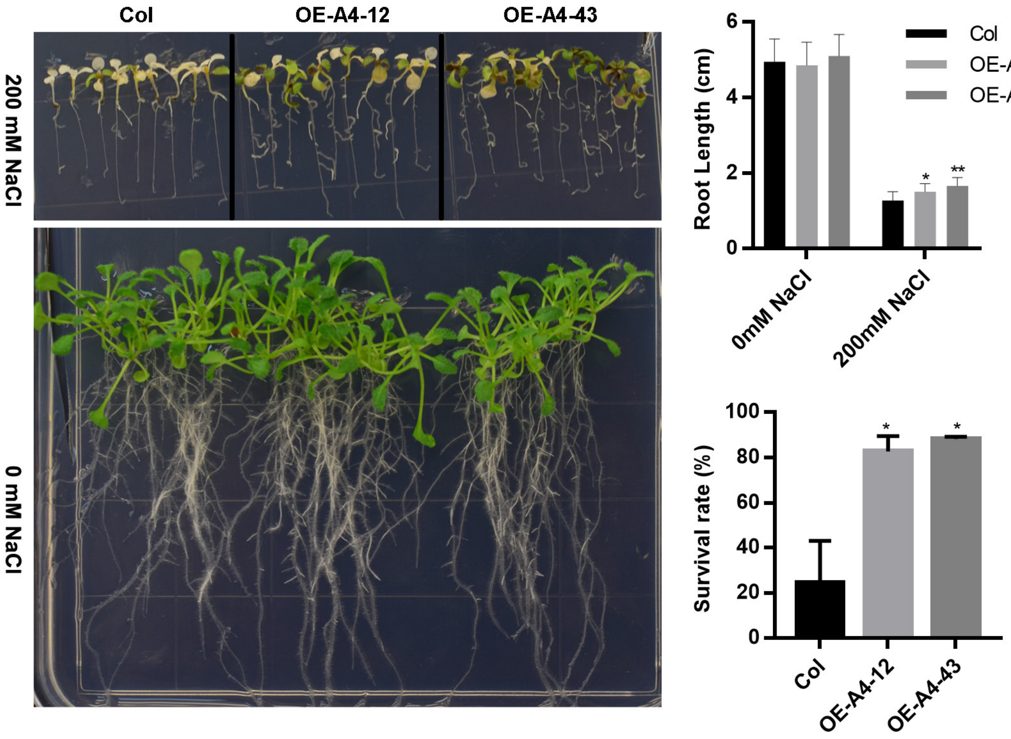

Supplementary Figure S3.Transgenic Arabidopsis plants of LlHsfA4 enhanced salt tolerance. 5-day old seedlings of two transgenic lines and wild type were transferred into MS medium containing 200 mM NaCl for 10 days before being photographed. T-test analysis of variance was employed to identify treatment means that differed statistically. Samples with different letters are significantly different: *p<0.05, **p<0.01.

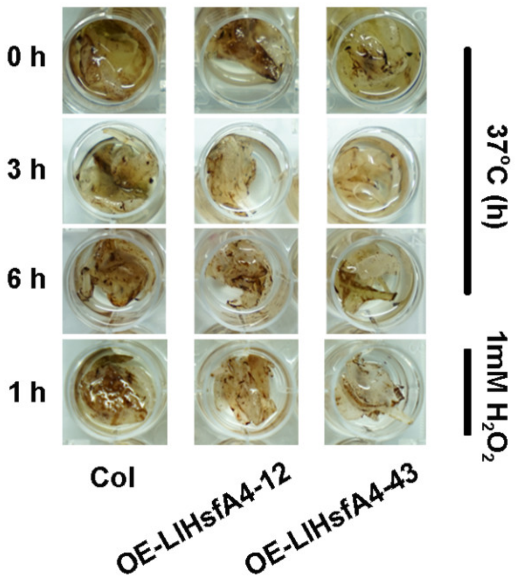

Supplementary Figure S4. Content of H_2O_2 decreased in transgenic Arabidopsis plants of LlHsfA4. 10-day old seedlings of two transgenic lines and wild type were treated with 1 mM H_2O_2 for 1 h or heat (37 °C) with different time and then used for DAB staining.

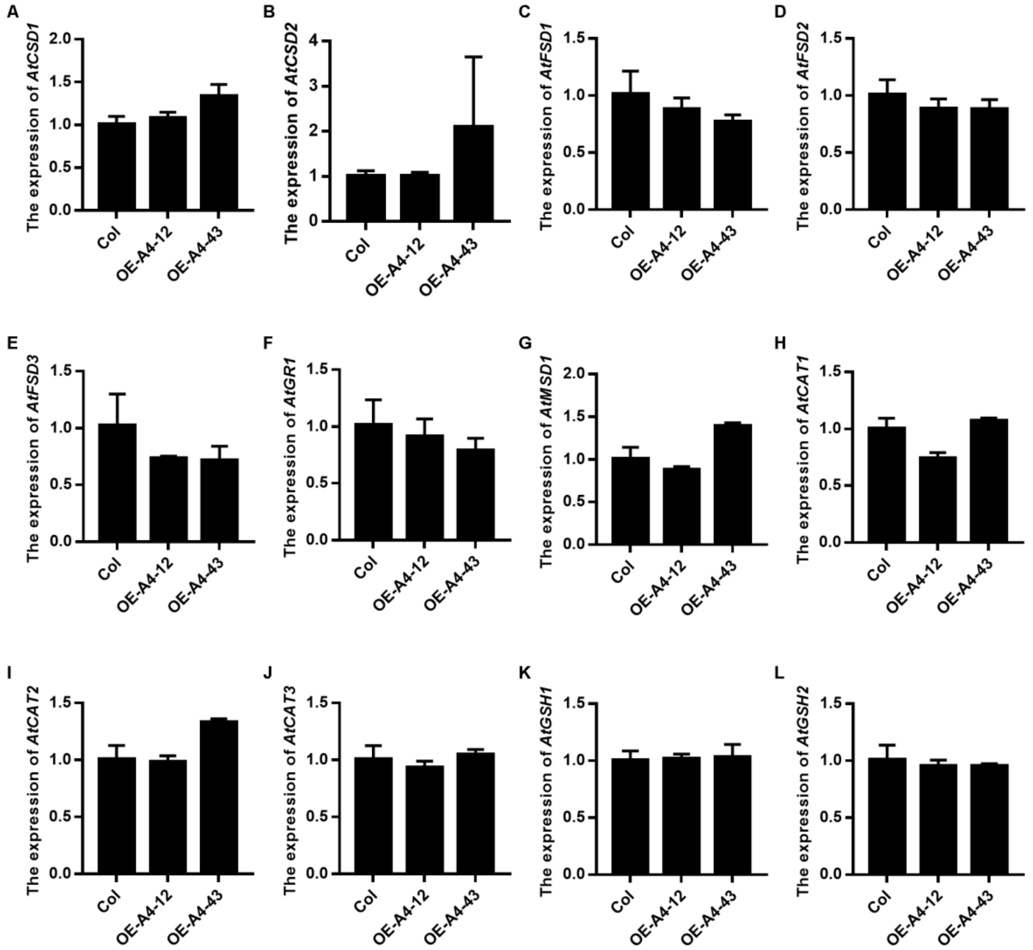

Supplementary Figure S5. Expression of genes related with ROS metabolism was examined in LlHsfA4 transgenic plants. The mRNA level of *AtSODs, AtCATs, AtGSH*, and *AtGRs* were detected in wild type (Col) and transgenic lines using qRT-PCR. Expression value of these genes in Col was setting as1 for comparison and *AtACTIN2* was used as an

internal standard. 10-day-old seedlings were collected for RNA extracting. Three independent experiments were performed each with three technical replicates and showing one experiment result.Unmarked means non-significance..

Table S1. Primers used in experiments.

Primer name	Primer sequence	Usage
LlHsfA4-Clone-F	5’TTYAAGCACARCAACTTCTCC 3’	Used for conserved sequence cloning
LlHsfA4-Clone-R	5’AGAAAYTGTTSCCARAABACATC 3’	
LlHsfA4-3’-F1	5’CAGAATCGTATGGAGACCTCTGTATCAGT 3’	3’RACE
LlHsfA4-3’-F2	5’CTTAATGAGCTTCGTGCCTCGTCTGAG 3’	
LlHsfA4-5’-R1	5’TCCAGTACCTGCATCTGCCGATC 3’	5’RACE
LlHsfA4-5’-R2	5’CTGCAGCTCGGATATCAAAGACCCC 3’	
LlHsfA4-F	5’ GAGTCAAACCTCCTCTCTGCTTCTTCTCC 3’	LlHsfA4 full-length cDNA clone
LlHsfA4-R	5’ GGAACAACATCAAGTCAGTACGCTAGAAAGG 3’	
LlHsfA4-SP1	5’ ATATACGTGTACACAATCCAACCTCACC 3’	Used for cloning of LlHsfA4 promoter.
LlHsfA4-SP2	5’ AGCAATCTGGCCAACTGAAGATGATTTAATAC 3’	
LlHsfA4-SP3	5’ CCACGTCAGAAATTCCAATAGAGATGATCCATG 3’	
LlHsfA4-F-*Xba*I-1300	5’ GCTCTAGAATGGACCCCTCGCAGGGGGC 3’	Used for *pSuper*::LlHsfA4-GFP vector construction.
LlHsfA4-R-*Kpn*I-1300	5’ GGGGTACCAGTTCTCTGTGCAGAAGCAAGATGCCC 3’	
LlHsfA4-F-*Pst*I-1391	5’ AACTGCAGTTGTAGATGACTTTGAGAACTTGTCTATAG 3’	Used for *pHsfA4*::GUS vector construction.
LlHsfA4-F-*Xma*I-1391	5’ TCCCCCGGGGGCGATCGGATCGATCGGAAC 3’	
LlHsfA4-PGBK-F-*Nde*I	5’ GGAATTCCATATGATGGACCCCTCGCAGGGGGC 3’	Used for pBD-LlHsfA4 vector construction.
LlHsfA4-PGBK-R-*Bam*HI	5’ CGGGATCCTTAAGTTCTCTGTGCAGAAGCAAGATGCCC 3’	

LlHsfA4-D1-R	5’ CGGGATCCTTACACCCCAGTGGTCGCAGC 3’	
LlHsfA4-D2-R	5’ CGGGATCCTTAGCTTTCAGCGTAATTCCTCGACTC 3’	
LlHsfA4-D3-R	5’ CGGGATCCTTACGAAGGCACACGAGGCAAG 3’	
LlHsfA4-D4-R	5’ CGGGATCCTTACATACGATTCTGACTCTCTGTATCAGC 3’	
LlHsfA4-RT-F	5’ CCGAGGGAGTATTTGGTGGAAC 3’	qPCR of *LlHsfA4*.
LlHsfA4-RT-R	5’ CTCAAAATAGATGGCCACAGAAGAAG 3’	
18S rRNA-F	5’ CTGAATCAGGATTGGATATCTGAGG 3’	qPCR of the *18S rRNA*.
18S rRNA-R	5’ AACTAGGTTACTGTCACTGGATAAC 3’	
AtActin2-F	5’ AGGAACTGGATCTGGTATGGGAACAT 3’	Primers used for qPCR
AtActin2-R	5’ GCAAATCCAGCCTTCACCAT 3’	
AtHsp17.6-F	5’ CCAAAGAAAAAGCCAAGAAGC 3’	
AtHsp17.6-R	5’ TGGAAACCTTCCAAACTCCA 3’	
AtZat6-F	5’ GTGACCTTGACCTGCCTTCTTC 3’	
AtZat6-R	5’ CTCCGGCAGATTGAGTAAGC 3’	
AtWRKY30-F	5’ AGAGCGATGATTCCGATCAAG 3’	
AtWRKY30-R	5’ CATCGTCCAGCGTTCTATCAA 3’	
AtMBF1c-F	5’ AGCAGATACCCAGGAGCAGT 3’	
AtMBF1c-R	5’ TTCGGATCGCGTAGGTCTTG 3’	
AtHsp25.3-F	5’ GATCAAGATGCGTTTCGACAT 3’	

AtHsp25.3-R	5’ TTCTACAGAGATTTTGACGTCTTCTT 3’
AtGolS1-F	5’ AGCCACCGGCTCTTTACTTC 3’
AtGolS1-R	5’ GTTCAGCGAAAGGAGTCGGA 3’
AtHsp22-F	5’ ACTACTCCAGGCAGCTTGCTA 3’
AtHsp22-R	5’ CTTGAATGGATCAGGGAACC 3’
AtZat12-F	5’ GACGCTTTGTCGTCTGGATT 3’
AtZat12-R	5’ GTGTCCTCCCAAAGCTTGTC 3’
AtAPX1 -F	5’ GTCCATTCGGAACAATGAGGTTTGAC 3’
AtAPX1 -R	5’ GTGGGCACCAGATAAAGCGACAAT 3’
AtAPX2 -F	5’ TGATGTGAAGACGAAGACAGGAGGAC 3’
AtAPX2 -R	5’ CCCATCCGACCAAACACATCTCTTA 3’
AtAPX3 -F	5’ CCCAAAATCACATACGCAGACCTGTA 3’
AtAPX3 -R	5’ AGTTGTCAAACTTCAGCGGCTCTTG 3’
AtAPX4 -F	5’ CTACTAAATCCGGGGGAGCCAATG 3’
AtAPX4 -R	5’ CTCTGTTGCATCACTCCTTCCAAAAT 3’
AtAPX5 -F	5’ AGCTAAACCGTCCACACAACAAAGGT 3’
AtAPX5 -R	5’ GTCCCAAAGTGTGACCTCCAGAGAGA 3’
AtAPX6 -F	5’ TGCAAAACGAAATAAGGAAAGTGGTG 3’
AtAPX6 -R	5’ CACTCAGGGTTTCTGGAGGTAGCTTG 3’

AtsAPX-F	5’ TGCTAATGCTGGTCTTGTGAATGCTT 3’
AtsAPX-R	5’ CCACTACGTTCTGGCCTAGATCTTCC 3’
AttAPX-F	5’ CAGAATGGGACTTGATGACAAGGAAA 3’
AttAPX-R	5’ ATGCAGCCACATCTTCAGCATACTTC 3’
AtCSD1 -F	5’ AGTAACCAAAGAGAGACGAAGCA 3’
AtCSD1 -R	5’ CCTTCCTGGGTGAAAAAGATAG 3’
AtCSD2 -F	5’ CGAAGGAGTTGTTACTTTGACCC 3’
AtCSD2 -R	5’ GAACCACAAAGGCTCTTCCAAC 3’
AtFSD1 -F	5’ CTCAAGCCACCTCCATTCG 3’
AtFSD1 -R	5’ GCGTTGTTGAAAGCAGGGA 3’
AtFSD2 -F	5’ TGGATTATCACTGGGGCAAAC 3’
AtFSD2 -R	5’ GGATAGACTCCCAGAAGAACTCG 3’
AtFSD3 -F	5’ GTGAACCCAACATCCCAATCG 3’
AtFSD3 -R	5’ TTGCGTCACTAACATTACTGTCACC 3’
AtGR1-F	5’ GGGTTTTGATGACGAAATGAGG 3’
AtGR1-R	5’ ATAGGACGACATCTGCCACGA 3’
AtMSD1-F	5’ GTTTGGGAGCACGCCTACTAC 3’
AtMSD1-R	5’ GTTCATCTCCTTATGTCATCGTGTA 3’
AtCAT1-F	5’ CGCCGATTTGCGAGATACA 3’

AtCAT1-R	5’ ACCCTCTCAGGAATCCGCTC 3’
AtCAT2-F	5’ TATCCAACTCCGCCTGCTGTCT 3’
AtCAT2-R	5’ ATGCGTGGGTCGGATAGGG 3’
AtCAT3-F	5’ GGTGACACTCAGAGACATCGCC 3’
AtCAT3-R	5’ AAACCTGTCTTGCCTGTCTGG 3’
AtGSH1- F	5’ TTTGAGCAGTATGTTGACTACGCAC 3’
AtGSH1- R	5’ GCAGTTCACCAGGGAGACAGG 3’
AtGSH2-F	5’ GGAAATGCTTTGCTGGGC 3’
AtGSH2-R	5’ TCTCCATAGATGTTGTTTCCTCC 3’
